# Role of nerve signal transduction and neuroimmune crosstalk in mediating the analgesic effects of acupuncture for neuropathic pain

**DOI:** 10.3389/fneur.2023.1093849

**Published:** 2023-01-23

**Authors:** Yong Chen, Dan Li, Ningcen Li, PeiYong Loh, Yi Guo, Xiyou Hu, Jingyu Zhang, Baomin Dou, Lifen Wang, Chaobo Yang, Tao Guo, Shuangli Chen, Zhen Liu, Bo Chen, Zelin Chen

**Affiliations:** ^1^Research Center of Experimental Acupuncture Science, Tianjin University of Traditional Chinese Medicine, Tianjin, China; ^2^School of Acupuncture & Moxibustion and Tuina, Tianjin University of Traditional Chinese Medicine, Tianjin, China; ^3^Laboratory Medicine, Nanfang Hospital, Southern Medical University, Guangzhou, China; ^4^School of International Education, Tianjin University of Traditional Chinese Medicine, Tianjin, China; ^5^National Clinical Research Center for Chinese Medicine Acupuncture and Moxibustion, First Teaching Hospital of Tianjin University of Traditional Chinese Medicine, Tianjin, China; ^6^School of Traditional Chinese Medicine, Tianjin University of Traditional Chinese Medicine, Tianjin, China

**Keywords:** acupuncture, analgesic effects, neuropathic pain, nerve signal transduction, neuroimmune crosstalk, metabolic and oxidative stress regulation

## Abstract

Neurogenic pain rises because of nervous system damage or dysfunction and is the most difficult to treat among other pathological pains. Acupuncture has been reported as a great treatment option for neurogenic pain owing to its unlimited advantages. However, previous studies on the analgesic effects of acupuncture for NP were scattered and did not form a whole. In this study, we first comprehensively review the relevant basic articles on acupuncture for NP published in the last 5 years and summarize the analgesic mechanisms of acupuncture in terms of nerve signaling, neuro-immune crosstalk, and metabolic and oxidative stress regulation. Acupuncture inhibits the upstream excitatory system and suppresses neuronal transmission efficiency by downregulating glutamate, NMDA receptors, P2XR, SP, CGRP, and other neurotransmitters and receptors in the spinal cord, as well as plasma channels such as TRPV1, HCN. It can also activate the downstream pain inhibitory pathway by upregulating opioid peptide (β-endorphin), MOR receptors, GABA and GABA receptors, bi-directional regulating 5-hydroxytryptamine (5-HT) and its receptors (upregulate 5-HT 1A and downregulate 5-HT7R) and stimulating hypothalamic appetite-modifying neurons. Moreover, neuroinflammation in pain can be inhibited by acupuncture through inhibiting JAK2/STAT3, PI3K/mTOR pathways, down regulating chemokine receptor CX3CR1 on microglia and up regulating adenosine receptor A1Rs on astrocytes, inhibiting the activation of glia and reducing TNF-α and other inflammatory substances. Acupuncture also inhibits neuronal glucose metabolism by downregulating mPFC's GLUT-3 and promotes metabolic alterations of the brain, thus exerting an analgesic effect. In conclusion, the regulation of nerve signal transduction and neuroimmune crosstalk at the peripheral and central levels mediates the analgesic effects of acupuncture for neuropathic pain in an integrated manner. These findings provide a reliable basis for better clinical application of acupuncture in the management of neuropathic pain.

## 1. Introduction

Neuropathic pain (NP) is chronic pain caused by a lesion or disease of the somatosensory nervous system (1). The pain may be spontaneous or evoked, as an increased response to a painful stimulus (hyperalgesia) or a painful response to a normally nonpainful stimulus (allodynia) (1). Functional, structural, and/or biochemical alterations in nerves are the basic cause of NP (2, 3). In addition to spontaneous pain, hyperalgesia and allodynia, the clinical manifestations of NP are often accompanied by symptoms such as anxiety and depression (2, 3). According to statistics, the prevalence of NP in the global population is about 7%−10% (4). Among various types of pathological pain, NP is considered the most difficult to cure. Despite the impressive progress in medicinal research in recent years, the existing pain management modalities are not ideal for NP. NP has high treatment costs and consumption of medical resources. Even more unfortunate are the adverse reactions of drugs that seriously affect patients' quality of life. The pathogenesis of NP is complex and much research has been published on the mechanisms associated with peripheral nerve, spinal cord, and neuraxial contraction levels in NP, but no single mechanism has been found that would explain all the symptoms of NP. Because many different mechanisms can explain the same symptoms, while during the development of NP, symptoms and their mechanisms are constantly changing. Studies suggest that acupuncture has great advantages in the treatment of NP and may be able to clinically relieve pain and improve the quality of life of NP patients (5).

Acupuncture has been used in China for more than 2000 years. With its rapid development, it has gained increasing recognition worldwide. Currently, it is being practiced in 183 countries and regions (6, 7). Of all the advantages associated with acupuncture, the analgesic effect is the most recognized. The analgesic effect of acupuncture is a comprehensive process that involves multiple pathways and levels, and is particularly associated with neuro-humoral regulation. Acupuncture achieves analgesia by promoting the endogenous opioid peptide release, upregulating local endorphins and peripheral opioid receptors during the inflammatory response while inhibiting endogenous pain-causing substances (8). Acupuncture interferes with the intracellular signaling pathways of spinal dorsal horn neurons, thereby producing analgesia (9). Electroacupuncture (EA) treatment can intervene in the early peripheral sensitization of NP by downregulating the phosphorylation level of transient receptor potential vanilloid receptor 1 (TRPV1) and calcitonin gene-related peptide (CGRP) expression in the dorsal root ganglion (DRG) (10). An investigation reported that EA modulates both small-diameter neurons TRPV1 and P2X3 purinoreceptor (P2X3) in the dorsal root ganglion (11). Acupuncture treatment can increase pain threshold, inhibit somatic pain, relieve acute and chronic pain, and reduce or even eliminate deep and involved pain along with emotional reactions to pain thereby improving patients' quality of life. Everyday new evidence confirms the analgesic effect of acupuncture on NP, this investigation summarizes the mechanisms associated with the analgesic effect of acupuncture in NP in terms of nerve signaling, neuro-immune crosstalk, and metabolic and oxidative stress regulation, to provide a reliable basis for better clinical acupuncture application for NP treatment.

## 2. Method

### 2.1. Retrieval strategy

Acupuncture is a method of stimulating acupoints or parts of the body with needles to prevent and treat disease. Electroacupuncture is a method in which needles are inserted into acupoints to Deqi, and then low-frequency pulse currents are introduced to stimulate and adjust the Qi of the meridians using both needle and electrical stimulation to prevent and treat diseases. Transcutanclus electrical acupoint stimulation (TEAS) is a method that combines transcutanclus electrical nerve stimulation (TENS) with acupoints to deliver specific low-frequency pulse currents through the skin to the body to treat pain. Acupuncture, Electroacupuncture and TEAS are the most commonly used acupuncture therapies for pain. Therefore, we chose them as the keywords for searching.

PubMed database was surveyed for the studies published from January 2017 to December 2021, by using the following keywords: [“acupuncture” or “electroacupuncture” or “transcutaneous electrical acupoint stimulation (TEAS)”] and [“analgesia” or “pain”]. The search formula used was: ((((acupuncture) OR (electroacupuncture)) OR (transcutaneous electrical nerve stimulation)) OR (TENS)) AND ((Analgesia) OR (pain)) AND (2017/1/1:2021/12/31[pdat]).

### 2.2. Research selection

**Figure 1:**
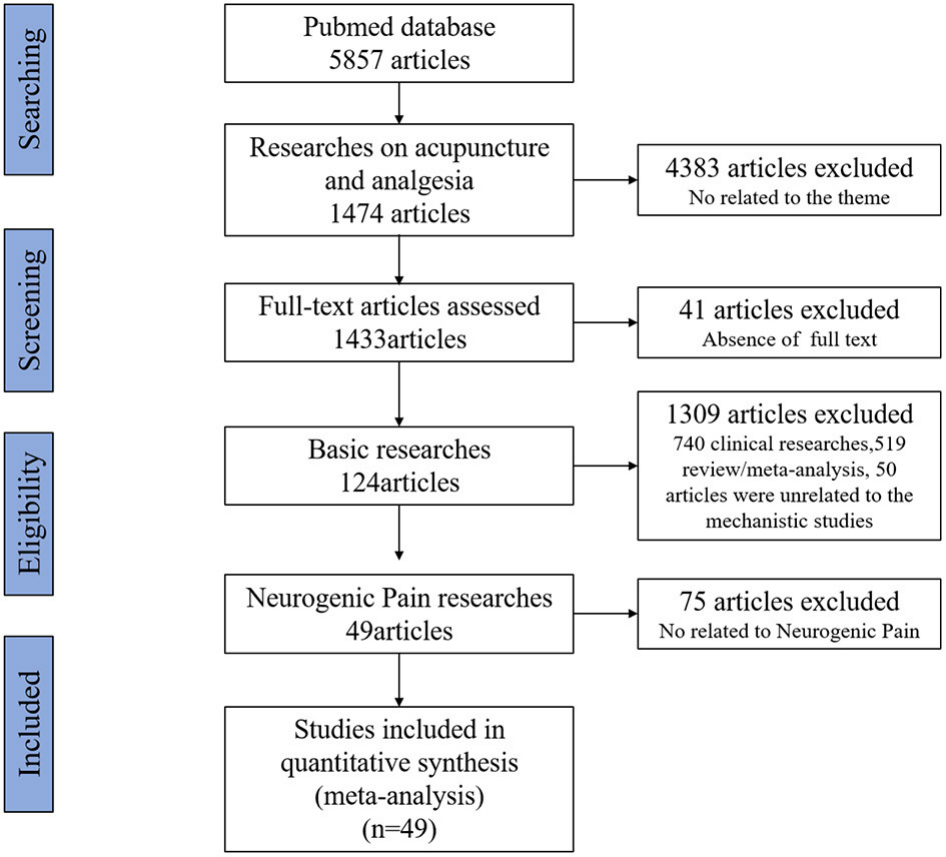
Flow chart of the retrieval strategy and process.

References that met the inclusion criteria were manually selected using Excel software before a close reading of the full text. Of 5,857 studies, 4,383 articles were not related to acupuncture and analgesia, 41 articles did not include full text, 740 were clinical research articles, 519 were reviews or meta-analyses, and 50 articles were not related to mechanistic studies, all of these were excluded. From the remaining 124 basic studies 75 articles were non-purely neuropathic pain such as inflammatory pain, cancer pain, and muscle or fascial pain were also excluded. In the end, 49 articles were selected for this review. The flow chart of the search process is shown in Figure 1.

### 2.3. Data extraction

For the mechanism studies, design data were extracted and classified using a predefined data extraction table that specified the type of neuropathic pain model, intervention (method, acupuncture point, acupuncture parameters), and outcome measures (pain-related behaviors, mechanism indicators). One author extracted the data and the other cross-checked it. Due to the similarity of some studies, the table enlists information only from some representative and recently published studies (Table 1).

**Table 1:** Modulation of analgesic effect of acupuncture on neurogenic pain.

**References**	**Pain model**	**Species**	**Intervention methods**	**Acupoints**	**Acupuncture parameter**	**Pain-related behavior**	**Test site**	**Biochemical measurements**
Jiang et al. (12)	CCI	Rat	EA	GB30/GB34	2 Hz, 1 mA, 30 min	PWT and PWL	Left mPFC	Glucose metabolism ↓ GLUT-3↓
Guo et al. (13)	CCI	Rat	EA/taVNS	ST36	15 Hz, 2 mA, 30 min	MWT and TWL	PFC, hippocampus, amygdala, hypothalamus	TNF-α↓
Yu et al. (14)	CCI	Rat	TEAS	GB34/GV26/ST36	6–9 mA, 2/100 Hz	MWT and TWL	DRG: L3–L5	MORs**↑**
Dai et al. (15)	CCI	Rat	EA	ST36	1 mA, 2/100 Hz, 30 min	MWT and TWL	L4–6 spinal cord	Adenosine↑, A1R↑
Zhang et al. (16)	CCI	Rat	EA	ST36/LR3	1 mA, 2/15 Hz, 30 min	MWT and TWL	L4-L6 spinal cord	GFAP↓, TNF-α↓
Chen et al. (17)	CCI	Mouse	MNS	PC6	2 Hz, 2 mA, 20 min	PWL	Hypothalamic	Orexin neurons↑
							vlPAG	OX1R↑, GABA↓
Tu et al. (18)	CCI	Rat	EA	ST36/GB34	2/100 Hz, 1.5 mA, 30 min	MWT and TWL	SCDH	BDNF↓,p-TrkB↓,TrkB↓, Iba1↓, activated microglia↓
Wang et al. (19)	CCI	Rat	EA	ST36/GB34	1 mA, 2/15 Hz, 30 min	MWT and TWL	L4–5 SCDH	Microgliacytes↓, astrocyte↓
Yang et al. (20)	CCI	Rat	EA	ST2/Jiachengjiang	2/100 Hz, 1–1.5–2 mA, 30 min	MWT and face-grooming	GG	HCN↓
Zhao et al. (21)	CCI	Rat	EA	ST36/SP6	2 Hz, 2 mA, 30 min	PWT	L4–5 spinal cord	NR2B↓
Li et al. (22)	CCI	Rat	EA	ST36/GB34	2/100 Hz, 1.5 mA, 30 min	MWT and TWL	L4–6 DRG	KCC2↑, GABAA↑
Huang et al. (23)	CCI	Rat	EA	GV20/GV14	2/15/50 Hz, 1 mA, 20 min	MWT and TWL	Hippocampus	GABAAR↑, GABA↓, Glutamate↓
							PAG	GABAAR↑, GABA↑
Jiang et al. (24)	CCI	Rat	EA	L4 and L6 Jiajixue	2/15 Hz, 2 mA, 15 min	MWT	Spinal cord	GABAAR↑
Wei et al. (25)	CCI	Mouse	EA	GB30/GB34	2 Hz, 1 mA, 30 min	MWT and TWL	Somatosensory cortex	Excitatory pyramidal neurons↓, GABAergic somatostatin-positive interneurons↑, vasoactive intestinal peptide-positive interneurons↓, CB1R↑
Du et al. (26)	SNI	Rat	EA	ST36/BL60	2/100 Hz, 0.5–1.0–1.5 mA, 30 min	PWT and PWL	DRG: L4–L6	P2X3↑ TRPV1↓
Wan et al. (27)	SNI	Rat	EA	ST36/SP6	2 Hz, 1–2–3 mA, 30 min	PWT and PWL	L1–L2 spinal cord	Syt-1↓
Wang et al. (28)	SNI	Rat	EA	ST36/SP6	2 Hz, 1–2–3 mA, 30 min	PWT	Spinal cord	IRF8↓, CX3CR1↓, IRF8&CD11b↓, CX3CR1 & CD11b↓
Wang et al. (29)	SNI	Rat	EA	ST36/SP6	2 Hz, 1–2–3 mA, 30 min	PWT	L4–L6 spinal cord	α7nAChR↑, IL-1β↓, CD11b↓
Wang et al. (30)	SNI	Rat	EA	ST36/SP6	2 Hz, 1–2–3 mA, 30 min	PWT	L4–L6 spinal cord	p-JAK2↓, p-STAT3↓, IL-6↓
Wang et al. (31)	SNI	Rat	EA	ST36/SP6	2 Hz, 0.5–1.5–2 mA, 30 min	PWL	Lateral hypothalamus	c-Fos-Positive Orexin Neurons↑
Zhu et al. (32)	SNI	Mouse	EA	ST36/SP6	2 Hz, 0.1 mA, 30 min	PWT	Brains	rACC Glu -vlPAG↓
Xia et al. (33)	SNI	Rat	EA	ST36/SP6	2 Hz, 1–2–3 mA, 30 min	PWT	L4–L6 spinal cord	HMGB1↓, TLR4↓, MyD88↓, NF-κB p65↓, CD11b↓
Ali et al. (34)	SNL	Rat	EA	ST36/SP6	2 Hz, 2–3 mA, 20 min	PWT	Spinal microglial	IL-10↑, β-endorphin↑
Wei et al. (35)	SNL	Rat	EA	GB30/GB34	2 Hz, 1–2–3 mA, 30 min	PWT	L5 DRGs	p-AXL↓, AXL↓
Zheng et al. (36)	SNL	Rat	EA	ST36/BL60	2/100 Hz, 1.5 mA, 30 min	MWT and TWL	L4–6 spinal cords	Iba-1↓, BDNF↓, P2X4↓, GABAAγ2↑
Liang et al. (37)	SNL	Rat	EA	ST36/BL60	2 Hz, 0.5–1.0–1.5 mA, 30 min	PWT and PFD	L4 DRG	P2X3R↓
Wu et al. (38)	SNL	Rat	EA	ST36/BL60	2 Hz, 1.5 mA, 30 min	MWT and TWL	L4–6 DRG	P2X7R↓, p-p38↓, Iba1↓ BDNF↓, IL-1β↓, IL-6↓, TNF-a↓, IL-10↑
Wang et al. (39)	SCI	Rat	EA	PC5/PC6	2 Hz, 2 mA, 20 min	PWT and PWL	L4–6 spinal dorsal horn	p-mTOR↓, p-S6K1↓, p-4E-BP1↓, SP↓, CGRP↓
Ji et al. (40)	SCI	Rat	EA	EX-B 2/BL25/BL40/BL60	1–2–3 mA, 2/100 Hz, 20 min	TWL	L4–L6 spinal cord	COX 2↓
Hou et al. (41)	BPAI	Rat	EA	T10–T12 Jiajixue	2/15 Hz, 30 min	TWL	SC, MC, Cpu, DLT	Metabolic alterations of brain↑
Xu et al. (42)	BPAI	Rat	EA	C5–C7 Jiajixue	2/15 Hz, 1.5 mA, 30 min	MWT and TWL	Spleen and lymph nodes	CD4+ T cells↑, β-endorphin↑, IL-17A↑, p-p65 NF-κB↑
Fei et al. (43)	DNP	Rat	EA	ST36/BL60	1 mA and 2 Hz	PWT and PWL	L4–6 DRGs	P2X3R↓
He et al. (44)	DNP	Rat	EA	ST36/BL60	2/100 Hz, 1–2 mA, 30 min	PWL	L4–L6 DRGs	P2X3 receptors↓, CGRP↓
Tang et al. (45)	DNP	Rat	MA	BL14/BL21/BL24	20 min	MWT and TWL	L4–6 DRG	P2X4↓, OX42↓
							Serum	CXCR3↓, TNF-α↓, IL-1β↓, IL-6↓, GSP↓, lipid metabolisms↓
Zhou et al. (46)	DNP	Rat	EA	ST36/BL60	2 Hz, 1 mA, 15 min	PWT	L4–L6 DRGs	P2X3R↓, p-PKC↓
Li et al. (47)	PHN	Rat	EA	GB30/GB34	2 Hz, 1 mA, 30 min	MWT	L4–L6 DRGs	Netrin-1↓, DCC↓, UNC5H2↑
Gao et al. (48)	Neck-incision pain model	Rat	EA	LI18, LI4-PC6, or ST36-GB34	2/100 Hz, 1 mA, 30 min	PT	C2–C5 dorsal cervicospinal cord	ATP↑, P2X7R↑, fractalkine↓, CX3CR1↓
Qiao et al. (49)	Neck-incision pain model	Rat	EA	LI18, LI4-PC6, or ST36-GB34	2/100 Hz, 1 mA, 30 min	PT	C3–C6 DRG	SP↓, CGRP↓, GAD67↑
Qiao et al. (50)	Neck-incision pain model	Rat	EA	LI18/LI4-PC6/ST36-GB34	2/100 Hz, 1 mA, 30 min	PT	C3–6 DRGs	SP↓, GFAP↓, GABA A α2R↑, GABA B R1↑
Wang et al. (51)	Neck-incision pain model	Rat	EA	LI18/LI4-PC6/ST36-GB34	2/100 Hz, 1 mA, 30 min	NWL	C2–C5 spinal cord	CB1↑, CB1R↑
Wang et al. (52)	Neck-incision pain model	Rat	EA	LI18/LI4-PC6/ST36-GB34	2/100 Hz, 1 mA, 30 min	NWL	C2–C5 spinal cord	GABA↑, GABA-AR↑, GABA-BR↑
Dai et al. (53)	Neck-incision pain model	Mouse	EA	SP6/GB34	1–2–3 mA, 30 min	PWT	L4–L6 spinal cord	IL-10↑, IL-10RA↑
Zhang et al. (54)	CIPN	Rat	EA	GB30	10 Hz, 2 mA, 30 min	MWT	Lumbar spinal cord	p-CaMKII↓, 5-HT 1A↑
Li et al. (55)	CIPN	Rat	EA	ST36/BL60	2 Hz, 0.5–1.5 mA, 30 min	PWT and PWL	L4–6 DRG	TLR4↓, MyD88↓, TRPV1↓, GFAP↓, OX42↓
Zhao et al. (56)	PINP	Rat	EA	ST36	10 Hz, 1 mA, 30 min	MWT	Spinal cord	TMEM119↓, GFAP↓, TLR4↓, NF-κB↓
Zhao et al. (57)	PINP	Rat	EA	PC5/PC6	2 Hz, 2 mA,20 min	PWT and PWL	DRGs (L4-L6)	Nrf2-ARE↑, SOD↑, NOX4↓, 8-iso PGF2α↓
Pei et al. (58)	Recurrent migraine	Rat	EA	GB20	2/15 Hz, 0.5–1.0 mA, 15 min	FMWT and HPL	Periaqueductal gray, raphe magnus nucleus, and trigeminal nucleus caudalis	5-HT7R↓
Liu et al. (59)	Recurrent migraine	Rat	EA	GB20/SJ17/GB34/ST36	2/15 Hz, 15 min	FMWT, PMWT, TFL, HPL and CPB	TG, TNC	5-HT7R↓, PKA↓, ERK1/2↓
Zhao et al. (60)	Recurrent migraine	Rat	EA	GB20	2/15 Hz, 0.5–1 mA, 15 min	FMWT and HPL	Trigeminal ganglion, trigeminal nucleus caudalis and ventroposterior medial thalamic nucleus	CGRP↓

**↑**, upregulated by acupuncture; **↓**, downregulated by acupuncture.

CCI, chronic constriction injury; PWT, paw withdrawal threshold; PWL, paw thermal withdrawal latency; mPFC, medial prefrontal cortex; GLUT-3, glucose transporter 3; MWT, mechanical withdrawal threshold; TWL, thermal withdrawal latency; PFC, prefrontal cortex; TNF-α, tumor necrosis factor-alpha; MORs, mu opioid receptors; A1R, adenosine A1 receptors; GFAP, glial fibrillary acidic protein; vlPAG, ventrolateral periaqueductal gray; OX1R, orexin 1 receptor; GABA, gamma-aminobutyric acid; SCDH, the dorsal horn of the spinal cord; BDNF, brain-derived neurotrophic factor; TrkB, tropomysin related kinase B; Iba1, ionized calcium binding adaptor molecule 1; GG, gasserian ganglion; HCN, hyperpolarization-activated cyclic nucleotide-gated; NR2B, N-methyl-D-aspartic acid receptor 2B; DRG, dorsal root ganglion; KCC2, K(+)-Cl(–) cotransporter 2; GABAA, gamma-aminobutyric acid subtype A; PAG, periaqueductal gray; GABAAR, gamma-aminobutyric acid type A receptors; CB1R, cannabinoid receptor 1; SNI, spared nerve injury; P2X3, P2X3 purinoreceptor; TRPV1, transient receptor potential vanilloid receptor 1; Syt-1, Synaptotagmin 1; IRF8, Interferon regulatory factor 8; CX3CR1, C-X3-C motif chemokine receptor 1; α7nAChR, alpha7 nicotinic acetylcholine receptor; IL-1β, interleukin-1beta; JAK2, Janus kinase 2; STAT3, signal transducer and activator of transcription 3; IL-6, Interleukin 6; rACC, rostral anterior cingulate cortex; HMGB1, high mobility group box-1; TLR4, Toll-like receptor 4; MyD88, myeloid differentiation primary response 88; NF-κB, noncanonical nuclear factor-kappaB; SNL, spinal nerve ligation; IL-10, interleukin 10; AXL, anexelekto; P2X4, P2X4 purinoreceptor; PFD, paw flinch durations; SCI, spinal cord injury; mTOR, mechanistic target of rapamycin; S6K1, S6 kinase 1; 4E-BP1, 4E-binding protein 1; SP, substance P; CGRP, calcitonin gene-related peptide; COX-2, cyclooxygenase-2; BPAI, brachial plexus avulsion injury; SC, somatosensory cortex; MC, motor cortex; Cpu, caudate putamen; DLT, dorsolateral thalamus; DNP, Diabetic neuropathic pain; GSP, glycated serum protein; PKC, protein kinase C; PHN, postherpetic neuralgia; DCC, deleted in colorectal cancer; UNC5H2, uncoordinated gene 5H2; PT, pain threshold; ATP, adenosine triphosphate; GAD67, glutamic acid decarboxylase 67; NWL, neck withdrawal latency; CIPN, chemotherapy-induced peripheral neuropathy; CaMKII, calcium/calmodulin-dependent protein kinase II; 5-HT 1A, 5-hydroxytryptamine 1A receptors; PINP, paclitaxel-induced neuropathic pain; TMEM119, transmembrane protein 119; Nrf2, nuclear factor E2-related factor 2; SOD, superoxide dismutase; NOX4, NADPH oxidase 4; 8-iso-PGF2α, 8-iso-prostaglandin F2alpha; FMWT, facial mechanical withdrawal threshold; HPL, hind-paw latency; PMWT, paw mechanical withdrawal threshold; CPB, cold-plate behaviors.

## 3. Animal models and intervention parameters of acupuncture for neuropathic pain

### 3.1. Animal models of acupuncture for neuropathic pain

NP can be divided into peripheral neuropathic pain, central neuropathic pain or mixed (peripheral and central) neuropathic pain, which is caused by damage to the peripheral or central nervous system. Commonly used animal models for NP include central nervous models such as spinal cord injury (SCI), spinal nerve ligation (SNL), and peripheral nervous models such as chronic constriction injury (CCI), spared nerve injury (SNI), brachial plexus avulsion injury (BPAI), diabetic neuropathic pain (DNP), postherpetic neuralgia (PHN), neck-incision pain, chemotherapy-induced peripheral neuropathy (CIPN) and recurrent migraine. These animal models provide a good guarantee for the smooth mechanism study of acupuncture in the treatment of NP.

### 3.2. Intervention parameters of acupuncture for neuropathic pain

**Figure 2:**
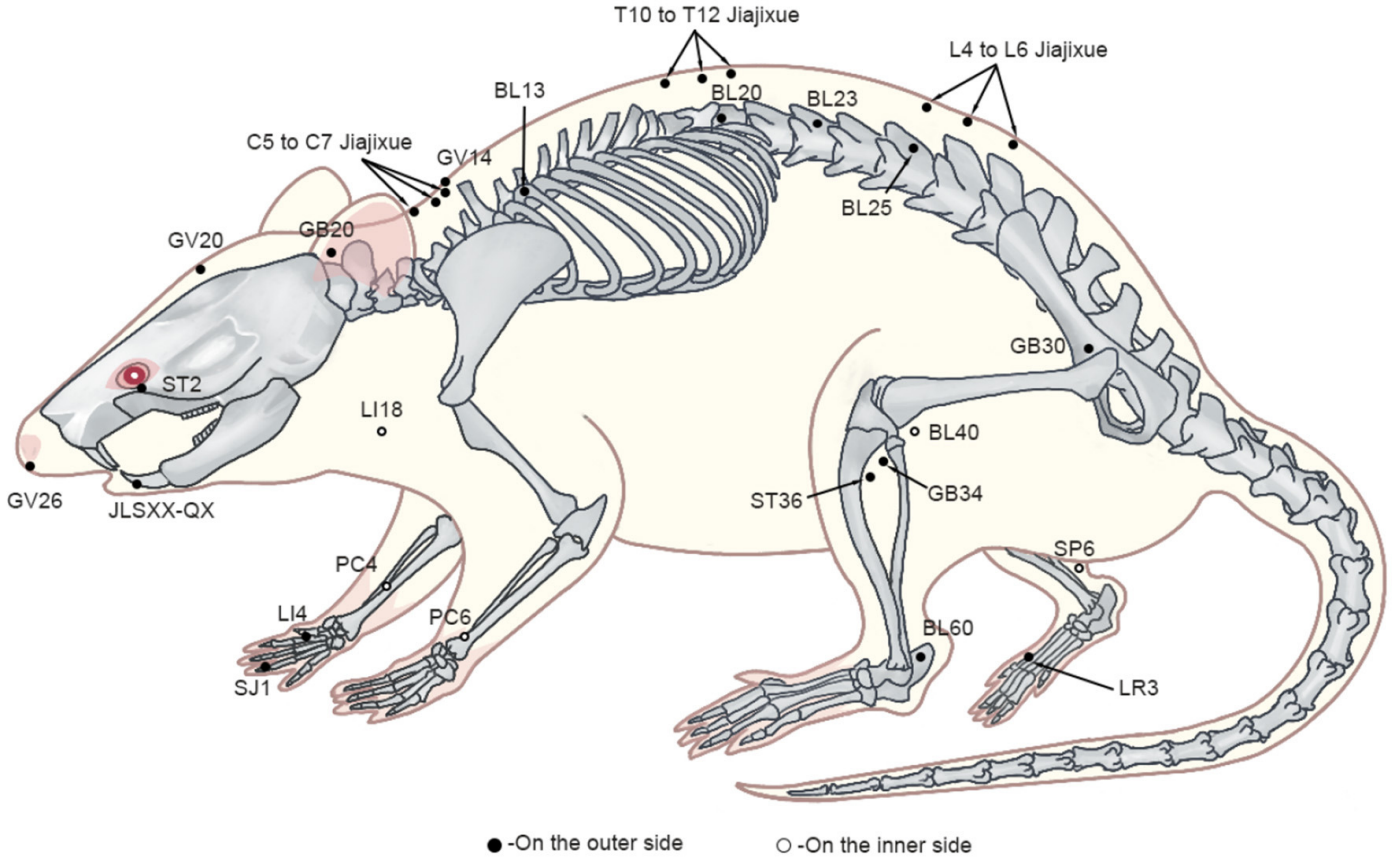
The schematic representation of acupoints.

Upon reviewing animal studies, it was noticed that 29 studies used ST36 (Zusanli) as the site of acupuncture intervention, 15 used GB34 (Yanglingquan), nine used BL60 (Kunlun), eight used SP6 (Sanyinjiao) and PC6 (Neiguan), five used GB30 (Huantiao) LI18 (Futu) and LI4 (Hegu), three used GB20 (Fengchi), two used PC4 (Ximen), one used GV26 (Shuigou), LR3 (Taichong), GV20 (Baihui), GV14 (Dazhui), SJ1 (Guanchong), BL13 (Feishu), BL20 (Pishu), BL23 (Shenshu), BL25 (Dachangshu), BL40 (Weizhong), ST2 (Sibai), JLSXX-QX (Jiachengjiang), C5–C7 Jiajixue, T10–T12 Jiajixue, L4 and L6 Jiajixue and L5 Jiajixue (Figure 2). These acupoints were selected mainly from the proximal and distal parts of the pain site, which is consistent with the principles of clinical acupuncture point selection. In EA, the following frequencies are most commonly applied: low (2, 10 Hz), high (100 Hz), and variable frequency (2/100, 2/15, 2/15/50 Hz), with the stimulation intensities of 0.5, 1, 1.5, 2, 3, 6–9 mA and stimulation times of 10, 15, 20, 30 min, with 30 min being the most frequently used stimulation time. The selection of EA parameters for animal models is similar to that applied in clinical treatments, which allows a better clinical translation of animal experimental results.

## 4. Mechanism of action of acupuncture in the treatment of neuropathic pain

When the organism is exposed to an external injurious stimulus, physical or chemical signals are transmitted to peripheral nerve endings and nociceptors. These nociceptors are primary afferent sensory neurons and consist mainly of myelinated Aδ fibers and unmyelinated C fibers (61). Excitatory information is transmitted through these fibers to the spinal dorsal horn (SDH), which activates nociceptive projection neurons in spinal cord lamina I *via* glutamate receptors (N-methyl-D-aspartic acid receptor, NMDAR), promotes inward flow of Na^+^ and Ca^2+^, subsequently carrying this information to the regions of the brainstem and brain that process pain sensory signals, thereby producing nociception (62, 63). Non-injurious stimulus signals are transmitted through the Aβ fibers of non-nociceptive receptors (64, 65) to the SDH region consisting of inhibitory interneurons, which contains inhibitory neurotransmitters such as gamma amino butyric acid (GABA), opioid peptides, endogenous cannabinoids, and 5-hydroxytryptamine (5-HT). This specific signal pathway leads to feedforward inhibition, thus ensuring that in a normal healthy state, Aβ fibers do not activate nociceptive projection neurons and can reduce their spontaneous activity, which is a key basis in the gating theory of pain transmission (66). However, in damaged neurons, signals in upstream excitatory and downstream inhibitory conduction systems can become aberrant, even leading to glial cell activation, causing inflammation in the peripheral nervous system (PNS) and central nervous system (CNS), further mediating the NP development (67–69).

### 4.1. Acupuncture inhibits the upward excitatory transmission system in neuropathic pain

When neurons are damaged, they will lead to the release of various factors such as glutamate, substance P (SP), calcitonin gene-related peptide (CGRP), adenosine triphosphate (ATP), etc. Primary injury sensing neurons transmit excitatory signals to the SDH and interact with their receptors, i.e., glutamate receptors (NMDAR, AMPAR, mGluR), SP receptors (NK1R), calcitonin receptor-like receptors (CRLR) and purinergic receptors (P2XR, P2YR), resulting in nociceptive hyperalgesia. These factors also alter the neuronal phenotypes, such that Aβ fibers (tactile neuronal fibers) shift from transmitting non-injurious signals to transmitting injurious signals; simultaneously abnormal neurons firing also affects surrounding non-damaged neurons, causing them to fire abnormally as well, thereby further transmitting injurious signals to the center.

Glutamate is the most common excitatory neurotransmitter in the CNS and plays a key role in the upstream excitatory transmission system with the following three receptors; NMDAR, α-amino-3-hydroxy-5-methyl-4-isoxazole-propionicacid receptor (AMPAR), and metabotropic glutamate receptors (mGluR) (70). Synaptotagmin 1 (Syt-1) is a synaptic vesicle protein which can regulate neurotransmitter release. In an SNI model, EA at bilateral ST36 and SP6 significantly reduced the expression of Syt-1 in the L1–L2 spinal cord, further inhibited neurotransmitters (such as glutamate) release, mediating analgesic effects of EA (27). The 2-Hz EA at ST36 and SP6 reduced glutamate in the rostral anterior cingulate cortex (rACC)-ventral lateral periaqueductal gray (vlPAG) loop in SNI mice model and revealed anxiety-like behavior and nociceptive hypersensitivity (32). In GV20 and GV14 EA in the CCI rat's model, hippocampal glutamate reduction was observed which produced an analgesic effect on NP (23). In addition, glutamate receptor expression plays an important role in EA analgesia. EA significantly reduces phosphorylation of calcium/calmodulin-dependent protein kinase II (CaMKII) in the spinal cord, inhibits voltage-dependent Ca^2+^ channels, and glutamate receptor NR2B (N-methyl-D-aspartic acid receptor 2B, NMDAR subunit) to reduce abnormal pain and nociceptive hyperalgesia in CIPN (54). Another study reported that EA at ST36 and SP6 acupoints in the CCI rats' model reduced NR2B in the L4–5 spinal cord, thereby exerting an analgesic effect (21). Thus, EA plays an analgesic role in NP by reducing glutamate release, inhibiting glutamate receptor expression, and suppressing the upstream excitatory system.

ATP not only provides energy to cells but also acts as a neurotransmitter in the PNS and CNS and is an important extracellular signaling molecule. In many pathological conditions, ATP is released from different cells and stimulates P2X/P2Y receptors and participate in pathological pain processes. P2X are ligand-gated cation channel receptors and P2Y receptors are the metabotropic receptors. P2X receptors bind to extracellular ATP, allow the permeation of cations such as Na^+^, K^+^, and Ca^2+^, cause depolarization of the membrane potential, and play an important role in the generation and transmission of information regarding injury (71). Each P2X receptor isoform has two transmembrane structural domains as well as a large glycosylated and disulfide-rich extracellular structural domain, with the ATP binding site located outside the cell. The homotrimer P2X3 receptor is mainly located in primary sensory neurons (small and medium-sized) which respond to injury and their peripheral nerve endings, suggesting that this receptor subtype is associated with the generation and transmission of nociceptive information (72). Studies have shown that the EA at ST36 and BL60 in SNL and DNP models can reduce P2X3 receptors in L4–6 DRGs (37, 43, 44, 46). EA also reduces P2X4 (36, 45) and P2X7 receptors (38) in L4–6 DRGs, inhibits excitatory postsynaptic potentials, neural loops excitability from the periphery to the spinal cord, abnormal dendritic/spinous synaptic reconstructions and inflammation to improve NP. EA modulates three main types of P2X receptors: the P2X3 receptors located in sensory neurons themselves, the P2X4 receptors located in microglia, and the P2X7 receptors located in glial cells (microglia, astrocytes, and oligodendrocytes) (73). Therefore, EA can regulate the function of neurons directly through P2X3Rs, or indirectly through glial neuron crosstalk by P2X4/P2X7Rs pathway. P2XR is mainly regulated in the DRGs.

SP and CGRP are neuropeptides released from sensory nerve endings and are the main mediators of neurogenic inflammation. In the neck-incision pain model, EA at acupoints: LI18, LI4-PC6, or ST36-GB34 decreases SP (49, 50) and CGRP (49), and at acupoint C3–6 it acts on DRGs to block the pain signals transmitted to the CNS. In a recurrent migraine model, EA at GB20 acupoint ameliorated migraine and associated abnormal cutaneous pain by reducing CGRP in the trigeminal ganglion (TG), the caudal nucleus of the trigeminal nucleus caudalis, and the ventroposterior medial thalamic nucleus of the thalamus (60).

Additionally, EA also reduces transient receptor potential ion channel vanilloid 1 (TRPV1) (26), anexelekto (AXL), p-AXL (35), and hyperpolarization-activated cyclic nucleotide-gated (HCN) in L4–6 DRGs (20) in gasserian ganglion (GG), which inhibits excitatory postsynaptic potentials and suppresses the upstream excitatory transmission system for analgesic effects.

All the above studies suggest that acupuncture produces analgesic effects by downregulating neurotransmitters and receptors such as glutamate and its NMDA receptors, P2XR, SP, CGRP, and ion channels (TRPV1 and HCN), which inhibit the upstream excitatory system and weaken the neuronal transmission efficiency.

### 4.2. Acupuncture promotes the descending inhibitory conduction system of neuropathic pain

When central inhibitory neurons die, they lose their inhibitory function and produce bursts of neuronal discharge, resulting in central NP. Decreased concentrations of inhibitory transmitters such as opioid peptides, GABA, and endogenous cannabinoids in the spinal cord and their reduced binding sites are also important causes of central NP.

Activation of opioid peptides (e.g., enkephalins, β-endorphins) and opioid receptors such as μ-opioid receptors (MOR) by acupuncture is a more recognized analgesic mechanism, which helps to inhibit the release of excitatory transmitters such as glutamate in the downstream pathway. EA suppresses NP by promoting the β-endorphins secretions from spinal microglia, spleen, and lymph nodes (34, 42). TEAS at GB34, GV26, and ST36 in CCI rats model upregulates the expression of MORs in L3-L5 DRGs (14), thereby activating the downstream inhibitory system. EA at GB30 and GB34 acupoints in PHN rats upregulates MORs and UNC5H2 in L4-L6 DRGs and downregulates DCC and Netrin-1, changes spinal cord dorsal horn's growth-permitting environment into an inhibitory environment, reducing RTX-induced primary afferent nerve sprouting, thus, acting as an analgesic (47).

GABA (inhibitory neurotransmitter) reduces pain perception through presynaptic inhibition (74). Studies have shown that EA promotes GABA and its receptors (GABA-AR, GABA-BR) in the spinal cord, DRG, and periaqueductal gray matter (PAG), inhibits excitatory neurotransmitters such as glutamate further reduces abnormal pain and nociceptive hyperalgesia in NP (22–24, 36, 50, 52). EA promotes the expression of glutamic acid decarboxylase 67 (GAD67) in C3-C6 DRGs of rats with neck-incision pain models. GAD67 up-regulates GABA expression and the upregulation of GABA inhibits excitatory postsynaptic potentials and reduces pain. Additionally, EA increases GABAergic somatostatin-positive interneurons, inhibits excitatory pyramidal neurons and vasoactive intestinal peptide-positive interneurons, and exerts analgesic effects which is dependent on the activation of endogenous cannabinoid receptor 1 (CB1R) (25). In neck-incision pain model rats, EA at LI18, LI4-PC6, and ST36-GB34 upregulates CB1 and CB1R in the C2–C5 spinal cord, and thus produces an analgesic effect (51). Alpha-7 nicotinic acetylcholine receptor (α7nAChR) is an ion channel receptor that is involved in the regulation of neurotransmitters such as GABA and 5-HT. It has been shown that EA upregulated α7nAChR in the L4–L6 spinal cord, thereby, upregulating GABA, 5-HT, etc. expression to reduce SNI-induced mechanical hypersensitivity (29).

5-HT is a very important neurotransmitter in living organisms. Under various psychological and physiological stresses, it can be accompanied by changes in synthesis and metabolism of 5-HT in the brain. Abnormal function of 5-HT in the CNS is closely related to pain, anxiety and cognition, and plays a biphasic regulatory role in the downward promotion and inhibition pathway. EA upregulates 5-HT 1A (54) and downregulates 5-HT7R to regulate PKA and ERK1/2 in the TG and trigeminal nucleus caudalis (TNC), mediating the antinociceptive hyperalgesic effects for migraine (58, 59).

Recent studies have shown that the reward effects of pain relief of EA analgesia are associated with the activation of hypothalamic appetite-modifying neurons (17, 31). EA activates appetitin neurons in the PAG, and the released appetitin then activates postsynaptic OX1Rs in the PAG, a Gq protein-coupled receptor that activates phospholipase C (PLC) to generate diacylglycerol (DAG), which can be converted to 2-arachidonic acid glycerol (2-AG; an endogenous cannabinoid, provided by DAGL). 2-AG then crosses the synapse in a retrograde manner and activates presynaptic CB1R, which stimulates the downstream pain inhibitory pathway, thus, producing analgesia. This disinhibition mechanism in vlPAG is mediated by the OX1R-PLC-DAGL-2-AG-CB1R cascade.

In conclusion, acupuncture upregulates opioid peptides such as β-endorphin and MOR receptors, GABA and its receptors, and bi-directionally regulates 5-HT and its receptors (upregulates 5-HT 1A and downregulates 5-HT7R) to activate the downstream pain inhibitory pathway and produce analgesic effects. Hypothalamic appetite-modifying neurons also play an important role in the analgesic effect produced by EA.

### 4.3. Acupuncture suppresses glial cell-mediated neuroinflammation

Neuro-inflammation is a critical cause of NP development, especially, PNS and CNS neuroinflammation caused by glial cell activation, which mediates the development and maintenance of NP (67–69). Microglia and astrocytes are the main immune cells of CNS, act as cell membrane receptors to respond to different neurotransmitters, interact with neurons to induce neuro-inflammation, and ease the transmission of pain signals. This response mechanism contributes to the adaptation of the CNS to changes in the internal environment mediated by injurious stimuli, leading to long-duration sensitivity and chronic pain in peripheral and central nociceptive neurotransmission pathways.

In the CCI model, EA can decrease central neuro-inflammation to reduce nociceptive hyperalgesia in NP by inhibiting TNF-α expression in the PFC, hippocampus, amygdala, and hypothalamus (13). The inhibition of JAK2/STAT3 (30) and PI3K/mTOR (39) pathways in spinal cord by EA plays an important role in the inhibition of central neuro-inflammation. In the CIPN model, EA inhibits L4–6 DRGs' TLR4 and MyD88 expression, TRPV1 expression, channel functional activity, and spinal glial cell activation (55). Acupuncture analgesia is mainly associated with the inhibition of microglia and astrocyte activation (19, 56). Microglia are the most important glial cells. EA downregulates microglial chemokine receptor (CX3CR1) expression, inhibits microglia activation, and reduces central neuro-inflammation pain by inhibiting TLR4/MyD88 co-expression and NF-κB p65 activation (18, 28, 33). Some findings suggest that inhibition of microglia activation by acupuncture may be associated with reduced cyclooxygenase-2 (COX-2) expression in spinal cord (40).

When astrocytes are active in the spinal cord segment, neuroinflammation can occur. EA can promote the upregulation of A1Rs on astrocytes and inhibits the active state of astrocytes, thus reducing the release of inflammatory substances such as GFAP and TNF-α, producing an analgesic effect (53). In the CCI model, EA at the ST36 acupoint increased the expression of L4–6 spinal adenosine and A1Rs (16), inhibited astrocytes activation, and played an important role in the analgesic effect for NP.

Therefore, EA inhibits the JAK2/STAT3 and PI3K/mTOR pathways, downregulates the microglial CX3CR1 and upregulates astrocytes A1Rs, inhibits glial cell activation, reduces inflammatory substances such as TNF-α, suppresses neuroinflammation, and thus has an analgesic effect.

### 4.4. The metabolic regulatory effects of acupuncture

Recently it has been revealed that the analgesic effect of acupuncture for NP may be related to metabolic regulation. EA at GB30 and GB34 significantly reversed increase in glucose metabolism induced by CCI and decreased the protein expression of glucose transporter 3 (GLUT-3) in the left medial Prefrontal Cortex (mPFC), suggesting that the analgesic effect of EA may be related to the inhibition of glucose metabolism and GLUT-3 expression in the mPFC (12). In a study involving the BPAI model, the brain metabolic connections between the bilateral hemisphere: somatosensory cortex (SC), the motor cortex (MC), caudate putamen (Cpu), and dorsolateral thalamus (DLT) were decreased compared with the normal animals. EA increased the strength of brain metabolic connections between the aforementioned regions at the 4th and 16th weeks, indicating that the regulation of brain metabolic connections may be an important mechanism that produces the analgesic effect by EA for the treatment of NP (41). Therefore, the mechanism of analgesic action of acupuncture in the treatment of NP may be elucidated from the perspective of metabolic regulation, and a new understanding may be obtained.

### 4.5. Oxidative stress regulation by acupuncture

When there is an oxidative/antioxidative imbalance, excess oxidative stress products can directly damage cell membrane structures, and organelles, and increase membrane permeability, leading to cellular dysfunction or even autolysis. A study investigated the analgesic effect of EA on paclitaxel-induced NP and found that under oxidative stress, EA upregulated the expression of Nrf2 antioxidant response element (Nrf2-ARE) and superoxide dismutase in the DRG, enhanced antioxidant signaling pathways, and inhibited oxidative stress products NOX4 and 8-iso-prostaglandin F2alpha (8-iso PGF2α), thereby attenuating paclitaxel-induced NP (57). Therefore, the regulation of EA on the level of oxidative stress of neurons may also be an important way to inhibit NP.

## 5. Conclusion

**Figure 3:**
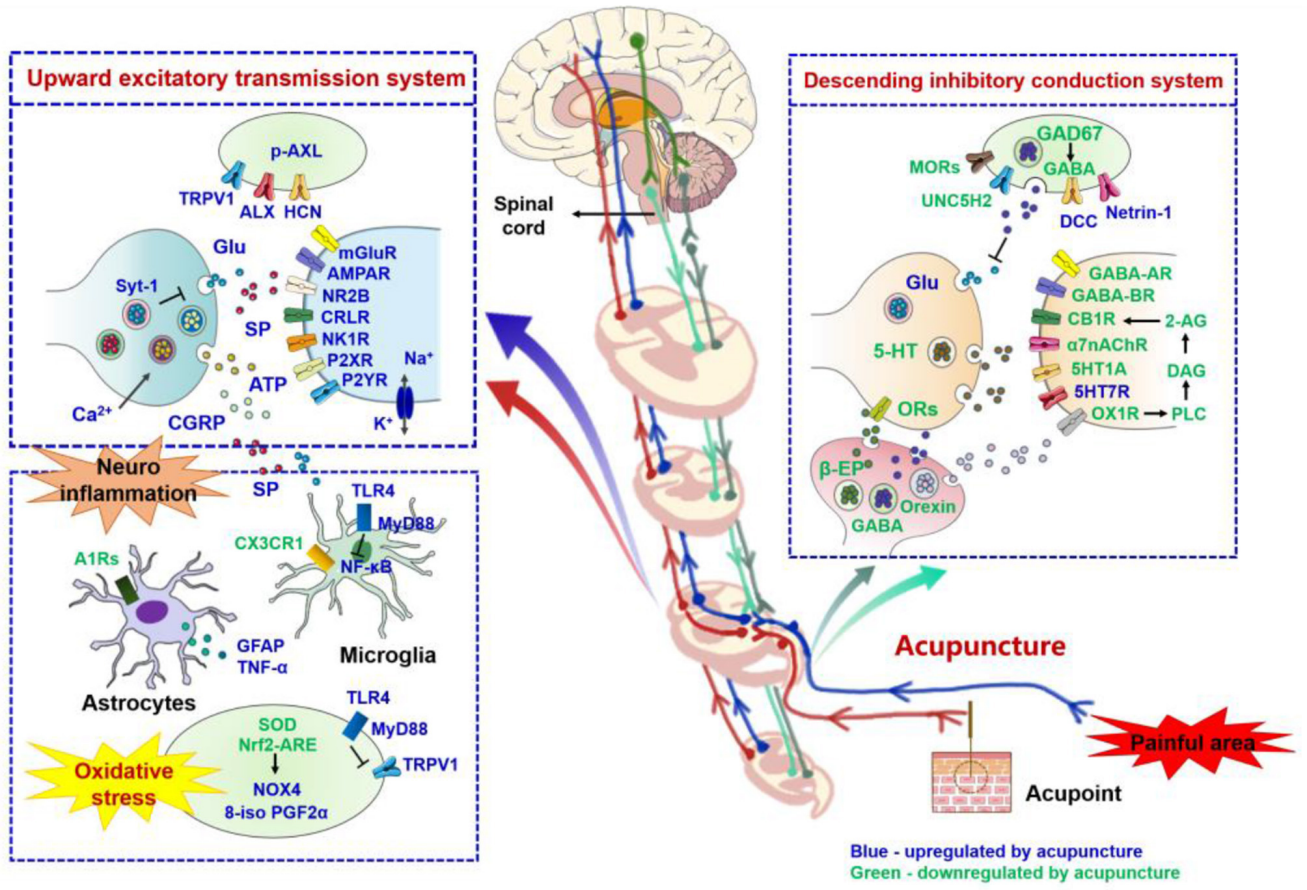
Role of nerve signal transduction and neuroimmune crosstalk in mediating the analgesic effects of acupuncture for neuropathic pain. The names of neurotransmitters, neuropeptides, and immune factors are presented in blue and green, respectively. The factors in red are up-regulated by acupuncture, while those in green are down-regulated by acupuncture. p-AXL, phosphorylation of anexelekto; AXL, anexelekto; TRPV1, transient receptor potential vanilloid receptor 1; HCN, hyperpolarization-activated cyclic nucleotide-gated; Glu, glutamate; Syt-1, synaptotagmin 1; mGluR, metabotropic glutamate receptors; AMPAR, α-amino-3-hydroxy-5-methyl-4-isoxazole-propionicacid receptor; NR2B, N-methyl-D-aspartic acid receptor 2B; CRLR, calcitonin receptor like receptor; NK1R, neurokinin 1 receptor; P2XR, P2X receptor; P2YR, P2Y receptor; SP, substance P; ATP, adenosine triphosphate; CGRP, calcitonin gene-related peptide; TLR4, toll-like receptor 4; MyD88, myeloid differentiation primary response 88; NF-κB, noncanonical nuclear factor-kappaB; A1Rs, adenosine A1 receptors; GFAP, Glial fibrillary acidic protein; TNF-α, tumor necrosis factor-alpha; CX3CR1, C-X3-C motif chemokine receptor 1; SOD, superoxide dismutase; Nrf2 - ARE, nuclear factor E2-related factor 2 -antioxidant response element; NOX4, NADPH oxidase 4; 8-iso-PGF2α, 8-iso-prostaglandin F2alpha; GAD67, glutamic acid decarboxylase 67; GABA, gamma-aminobutyric acid; MORs, mu opioid receptors; A1R, adenosine A1 receptors; UNC5H2, uncoordinated gene 5H2; DCC, deleted in colorectal cancer; 5-HT, 5-hydroxytryptamine; CB1R, cannabinoid receptor 1; 2-AG, 2-arachidonic acid glycerol; DAG, generate diacylglycerol; PLC, phospholipase C; OX1R, orexin 1 receptor; α7nAChR, alpha7 nicotinic acetylcholine receptor; β-EP, beta-endorphin; ORs, opioid peptide receptors.

In NP models, EA inactivates the upstream excitatory system and reduces the neuronal transmission efficiency by inhibiting neurotransmitters and receptors such as glutamate, NMDA receptors, P2XR, SP, CGRP, and ion channels (TRPV1 and HCN). It also enhances opioid peptides such as β-endorphin and MOR receptors, GABA and its receptors, bi-directionally modulates 5-HT and its receptors (upregulate 5-HT 1A and downregulate 5-HT7R), activates hypothalamic appetitin neurons, and thus, stimulates the downstream pathway for pain inhibition. Moreover, EA can inhibit the occurrence of neuroinflammation to produce analgesic effect by inhibiting JAK2/STAT3, PI3K/mTOR pathways and neuroglial cell activation, down regulating CX3CR1 in microglial and inflammatory substances (TNF-α), up regulating adenosine receptor A1Rs on astrocytes. Neuronal glucose metabolism inhibition by down-regulating of mPFC GLUT-3 expression and increased cerebral metabolic connections between SC, MC, Cpu, and DLT were also associated with analgesic effects produced by EA. Additionally, neuronal oxidative stress regulation by EA may also be an important pathway associated with decreased NP. In conclusion, according to existing research, EA inhibits the occurrence and development of NP by inhibiting the upward excitatory transmission system, promoting the descending inhibitory conduction system, suppressing glial cell-mediated neuroinflammation, and regulating the level of metabolism and oxidative stress (Figure 3). These elucidations of the analgesic mechanisms of EA provide scientific evidence for the treatment of NP with EA, which may help reduce the analgesic drug dosage, drug-induced side effects, and break through the bottleneck problem of clinical treatment options for NP.

## Author contributions

YC, DL, and NL contributed to the concept design, data collection, and paper writing. LW, CY, and TG contributed to the figures and graphic abstract edit. XH, JZ, SC, and ZL contributed to the data collection and analysis. BD, PL, and YG contributed to the language modification and text check. BC and ZC contributed to the concept design and paper review. All authors contributed to the article and approved the submitted version.
